# Experimental Study on Dynamic Characteristics of Damaged Post-Tensioning Concrete Sleepers Using Impact Hammer

**DOI:** 10.3390/ma17071581

**Published:** 2024-03-29

**Authors:** Jung-Youl Choi, Tae-Hyung Shin, Sun-Hee Kim, Jee-Seung Chung

**Affiliations:** 1 Department of Construction Engineering, Dongyang University, No. 145 Dongyangdae-ro, Punggi-eup, Yeongju-si 36040, Gyeongsangbuk-do, Republic of Korea; jychoi@dyu.ac.kr (J.-Y.C.); jschung@dyu.ac.kr (J.-S.C.); 2 Engineering HQ, Seoul Metro, 5, Hyoryeong-ro, Seocho-gu, Seoul 06693, Republic of Korea; sss0930@seoulmetro.co.kr; 3 Department of Architectural Engineering, Gachon University, 1342 Seongnamdaero, Sujeong-gu, Seongnam-si 13120, Gyeonggi-do, Republic of Korea

**Keywords:** concrete sleepers, impact hammer test, natural frequency, cracks, numerical analysis

## Abstract

Concrete sleepers in operation are commonly damaged by various internal and external factors, such as poor materials, manufacturing defects, poor construction, environmental factors, and repeated loads and driving characteristics of trains; these factors affect the vibration response, mode shape, and natural frequency of damaged concrete sleepers. However, current standards in South Korea require only a subjective visual inspection of concrete sleepers to determine the damage degree and necessity of repair or replacement. In this study, an impact hammer test was performed on concrete sleepers installed on the operating lines of urban railroads to assess the field applicability of the modal test method, with the results indicating that the natural frequency due to concrete sleeper damage was lower than that of the undamaged state. Furthermore, the discrepancy between the simulated and measured natural frequencies of the undamaged concrete sleeper was approximately 1.87%, validating the numerical analysis result. The natural frequency of the damaged concrete sleepers was lower than that of the undamaged concrete sleeper, and cracks in both the concrete sleeper core and the rail seat had the lowest natural frequency among all the damage categories. Therefore, the damage degrees of concrete sleepers can be quantitatively estimated using measured natural-frequency values.

## 1. Introduction

Concrete sleepers on railroad tracks can be damaged because of repeated loads and driving characteristics of trains, resulting in the cracking and dislodging of tie bar contacts in the interior of the sleeper, tie bar breakage, breaking around fasteners, and fissures in the concrete underneath the rail. Damage to concrete structures can affect their material and structural properties, such as dynamic responsiveness, natural frequency, mode forms, and damping ratio. Nonetheless, according to South Korean standards [1], the condition of damaged sleepers and the necessity of repair or replacement are determined by a subjective visual inspection of the damaged sleeper. Additionally, damaged concrete sleepers must be removed from the railway, making the entire inspection process time-consuming and expensive.

In South Korea, sleeper inspection is performed using two methods: visual inspections and rebound hardness tests. In visual inspections, the sleeper’s damage is judged subjectively by the inspector via a qualitative appraisal. According to Clark et al. [2], the increasing demand on track structures creates a need for an appropriate monitoring system. Visual observation methods do not record damage in real time and do not have sufficient efficiency to meet the significant demand for reduced track possessions. By applying non-destructive technologies (NDTs), the risk due to structural damage to the track can be reduced. As a result of monitoring the structural condition of the damaged structure through acoustic emission, it was confirmed that it was effective in detecting early cracks.

The rebound hardness test examines the material strength of the concrete but does not assess the structural integrity of damaged concrete sleepers. Due to the limitations of these two methods, an improved method is required to determine the condition of concrete sleepers.

To better understand the structural behavior of damaged concrete sleepers, Choi [3] conducted experimental and computational assessments of the changes in the dynamic characteristics of concrete sleepers via an impact hammer test. Kim and Jung [4] performed an impact hammer test to assess the structural integrity of old railroad plate girder bridges. These researchers also performed a mode analysis of the full-scale field experiments to verify their reliability. Esmaeili et al. [5] tested the dynamic resistance of concrete, wood, and steel sleepers under lateral impact loading conditions in the laboratory, with steel sleepers found to have insufficient dynamic resistance to impact loads compared with concrete and wooden sleepers.

Braunfelds et al. [6] proposed a technology that can measure read deformation over a long period of time using an optical FBG sensor. Chung et al. conducted field applicability evaluation using the rebound hardness test method to estimate the strength of sleeper floating track concrete sleepers. As a result, the strength estimation results showed differences depending on the sleeper’s condition and support conditions [7].

Choi et al. [8] estimated the spring stiffness of the sleeper floating track sleeper resilience pads using finite element analysis. The vibration acceleration of the ballast in the numerical analysis was within the range of field measurement results.

Sapidis et al. [9] proposed autonomous compression damage of fiber-reinforced concrete using a piezoelectric lead zirconate titanate transducer using a 1D convolutional neural network. As a result, it showed an accuracy of 95.25% with the experimental results.

Nielsen and Palmer [10] discovered that the modal analysis of prestressed concrete sleepers was useful for identifying dynamic behavior and reaction, with the modal analysis results represented as frequency response functions (FRFs) for various sleeper states. Through finite element analysis, Kaewunruen and Remennikov [11] analyzed the sensitivity effect on the free-vibration characteristics of concrete sleepers in the field caused by the changes in the stiffness and material properties of the rail pads using Timoshenko beam and spring elements. The finite element analysis results confirmed that the rail pad parameters have a nonlinear effect on the effective stiffness, influencing the field track system and significantly affecting the frequency and mode shape of additional modes. Kaewunruen and Remennikov [12] performed an impact hammer test, a nondestructive test method, to evaluate the structural integrity of a gravel road track, and they compared the time–acceleration response function from field measurements with the visual inspection results. By performing impact hammer experiments on recovered damaged concrete sleepers, Shin [13] proved that damage to sleepers can reduce the stiffness of sleepers but has minimal effects on their mass-and-damping ratio. Furthermore, it was proven experimentally and analytically via comparison with numerical analysis results that the natural frequency of damaged sleepers is lower than that of undamaged sleepers, depending on the extent of the damage. Finally, the vibration of the sleeper was examined by Lam and Wong [14] through an impact hammer test to determine whether the sleeper was damaged. Kaewunruen and Remennikov [15] used modal analysis to evaluate the modal change in vibration characteristics of prestressed concrete sleepers in the 0–1600 Hz frequency band. The experiment evaluated the impacted specimen with a drop impact tester. The modal parameters of undamaged sleepers and cracked sleepers were compared.

You et al. summarized the damage patterns of prestressed concrete sleepers to provide better insight into the damage effects of railway-reinforced concrete sleepers so that they could be used to improve track maintenance and inspection criteria. They analyzed the theory of the causes of prestressed concrete sleepers based on the limit state method [16].

Real et al. investigated the dynamic effect of cracks on the vibration characteristics of railway-prestressed concrete sleepers to identify structural defects. They highlighted changes in the modal parameters of healthy and cracked sleepers in terms of natural frequencies and modal damping [17]. Matsuoka and Watanabe applied a frequency-based detection method to evaluate damaged concrete sleepers. As the frequency-based damage-detection method, which has limited practical application, was used, damage to concrete was confirmed through experiments to validate its feasibility [18].

According to the results of previous studies, we propose the impact hammer test, a modal test technique, as a quantitative method for evaluating the structural soundness of concrete sleepers. The impact hammer test was employed to compare field data with analytical results. A post-tensioned sleeper with rail support points buried in the concrete road surface at both ends of the sleeper was used, and the sleeper floating track was elasticized by inserting resilience pads at the corresponding positions. The dynamic response changes of concrete sleepers induced by the post-tensioning method were analyzed via field measurements and finite element analysis to compare the damage to concrete sleepers and the dynamic characteristic changes using the impact hammer test. Furthermore, field measurements were performed on a public urban railroad’s sleeper floating track to examine the effect of the damage degree of concrete sleepers on the natural frequency change. The impact hammer test is a portable, easy-to-apply method for maintaining concrete sleepers.

## 2. Materials and Methods

### 2.1. Measurement Sections and System

Field measurements were performed on concrete sleepers of antivibration track structures built on urban railroad lines in Korea. The antivibration track structure was used on steeply curved concrete sleepers with a curve radius (R) of less than 400 m and is one of the sleeper floating orbits with a resilience pad placed on the bottom. The measuring track comprised a concrete track in a tunnel with a curve radius of 250 m (cant: 110 mm), a vibration-reducing track structure, and a train speed of 50 km/h.

**Figure 1:**
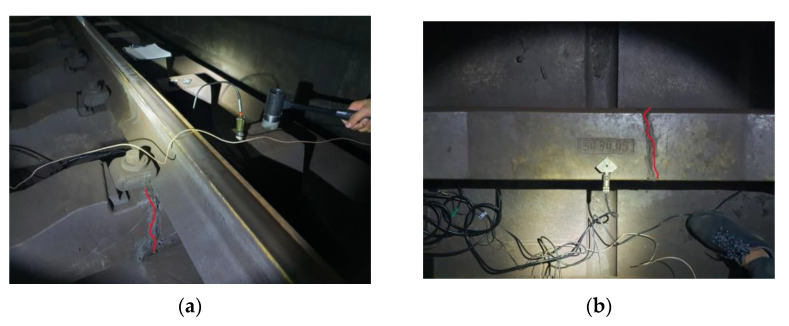

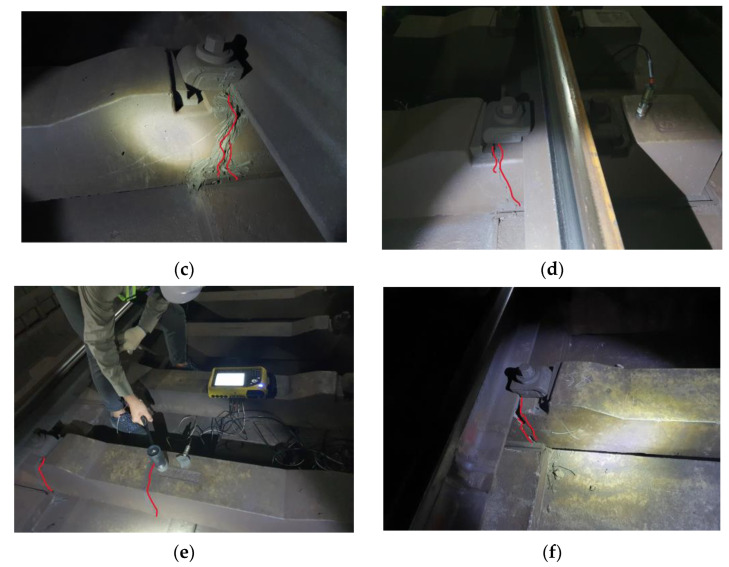
Visual inspection of concrete sleepers. (**a**) Crack #1 (rail seat); (**b**) Crack #1 (center); (**c**) Crack #2 (rail seat); (**d**) Crack #2 (rail seat); (**e**) Crack #3 (rail seat, center); (**f**) Crack #3 (rail seat).

Impact hammer tests were performed on one undamaged concrete sleeper and three damaged concrete sleepers with cracks in the sleeper center and rail seat based on visual inspection. The impact hammer test is a method of evaluating the dynamic properties of concrete through the impact of the hammer and the response signal measured with an accelerometer. To check the stiffness of concrete, the influence of dynamic characteristics must be checked. Acceleration gauges are installed inside and outside of track gauge. For the damaged concrete sleepers, the damage was concentrated on the rail seat and sleeper center, as shown in Figure 1. Figure 1a,b,d,f shows the damage caused to the sleeper directly below the rail. Figure 1e shows damage to the sleeper directly below the rail; here, damage occurred in the center of the sleeper.

**Figure 2:**
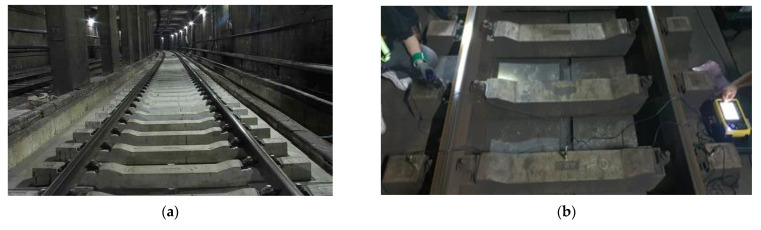

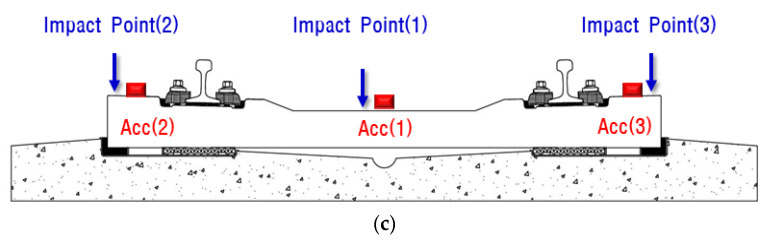
Photographs of modal test using impact hammer. (**a**) Overview of test track; (**b**) impact hammer test; (**c**) schematic of instrumentation.

The field measurement section is shown in Figure 2a, and accelerometers were placed on the top surface of the sleeper to measure the vertical acceleration of the sleeper during the impact hammer blow, as shown in Figure 2b,c. Table 1 shows the sensitivity of each sensor of the impact hammer and accelerometer employed in the measuring method. The dynamic response (acceleration) measurement data against the load created by the impact hammer blow were used to study the frequency response function (FRF). The FRF allows us to calculate the characteristics of dynamic responses in the frequency domain and the relationship between mass and stiffness, which is expressed as the ratio of the dynamic response to the impact load.

### 2.2. Dynamic Mass-and-Stiffness Estimation Using FRF

The relationship between the loads acting on a structure and its dynamic reaction is critical for determining its dynamic features. This reaction is known as an FRF in the frequency domain. The FRF can be used to estimate values above the natural frequency for dynamic masses and below the natural frequency for dynamic stiffness.

The vibration response of a structure can be used to generate several forms of FRFs. In this study, the standard FRF was used to determine the structure’s dynamic mass. 

The relationship between the applied load and dynamic response in the frequency domain is summarized in Table 2 [13]. In Table 2, F is the load and u is the displacement.

**Figure 3:**
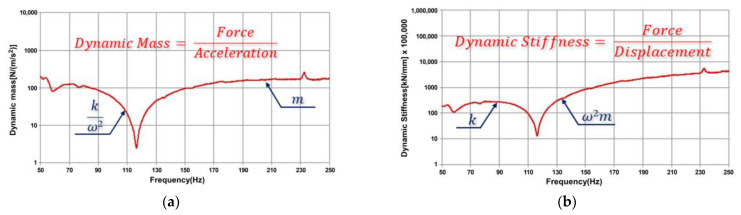
Dynamic mass-and-stiffness estimation of single degree of freedom. (**a**) Dynamic mass (m); (**b**) dynamic stiffness.

Through FRF, the dynamic mass was estimated in the range exceeding the natural frequency, as shown in Figure 3a. Dynamic stiffness was estimated below the natural frequency, as shown in Figure 3b.

Using FRF, the dynamic mass-and dynamic stiffness of a structure can be estimated, and the relationship is summarized in Table 3 [13].

### 2.3. Numerical Analysis

#### 2.3.1. Numerical Analysis Conditions

**Figure 4:**
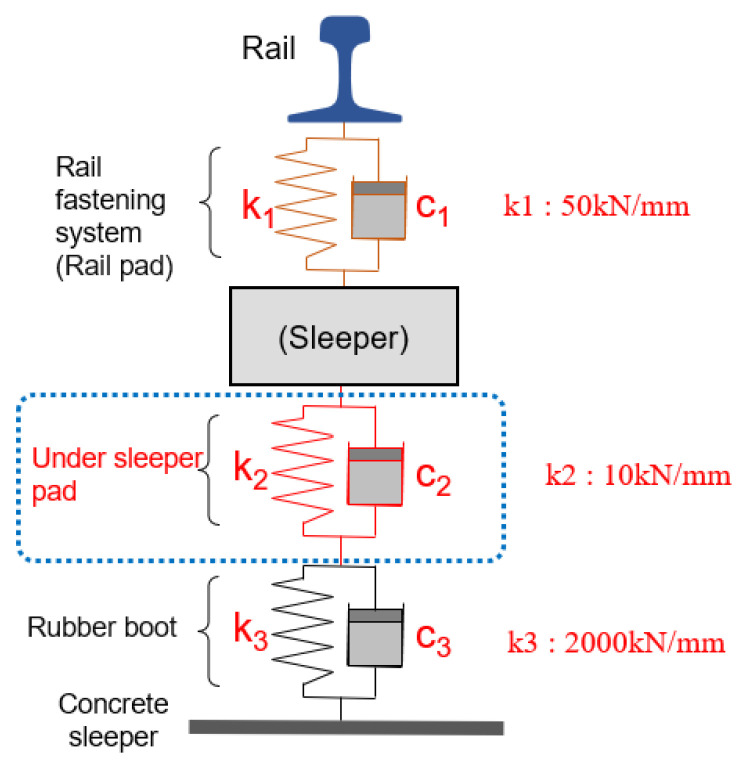
Concrete sleeper spring model.

The rail-connection mechanism and spring element of the sleeper floating track were used in the same manner as in the field. The internal steel bars of the post-tensioning sleeper were simulated, and tension forces for each steel bar were added, as shown in Figure 4. Self-weight and external-impact loads were considered in the numerical analyses. The impact load was 700 N, as determined via the impact hammer test. Rail pads (k_1_, 50 kN/mm) inserted into the rail seat, under sleeper pads (k_2_, 10 kN/mm) inserted into the lower section of the sleeper, and rubber boots (k_3_, 2000 kN/mm) were used as spring elements. The stiffness of the rail pad, under sleeper pad, and rubber boots used the values presented in Seoul Metro’s internal specifications.

#### 2.3.2. Modeling

**Figure 5:**
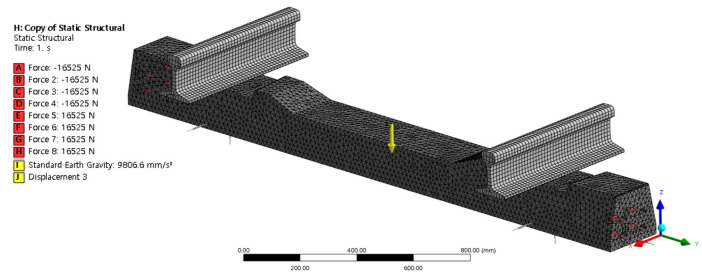
Post-tensioning concrete sleeper modeling.

Figure 5 shows a numerical model corresponding to the field-measured concrete sleeper in Section 2.1. A numerical analysis was performed using Ansys Workbench Ver. 2021 R2 [19] to analyze the natural frequency of the undamaged concrete sleepers. For the concrete sleepers, modal analysis was performed on a total of six modes supported by the Ansys program. Modal analysis is a method of analyzing the dynamic characteristics of a structure or the characteristics of a structure subjected to dynamic external forces. Dynamic characteristics include resonance frequency and mode shape attenuation. Each mode represents a free vibration phenomenon at the mode frequency, which is influenced by the weight and rigidity of the structure.

In general, simple structures can be said to have the first mode shape in the most flexible direction. In the case of a cantilever with one side fixed, the first mode shape can be predicted because the largest deformation is at the opposite end.

The nodes and elements were calculated automatically during their generation in the ANSYS Workbench program. The mesh of the sleeper contained 248,777 nodes and 148,707 elements, with the element size of the sleeper assumed to be 20 mm. The size of the steel bar element was 10 mm and that of the rail element was 15 mm. Table 4 presents the specifications of the analysis model.

## 3. Results and Discussion

### 3.1. FRF Results

**Figure 6:**
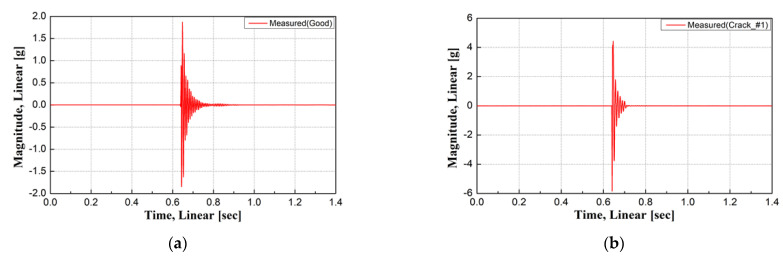

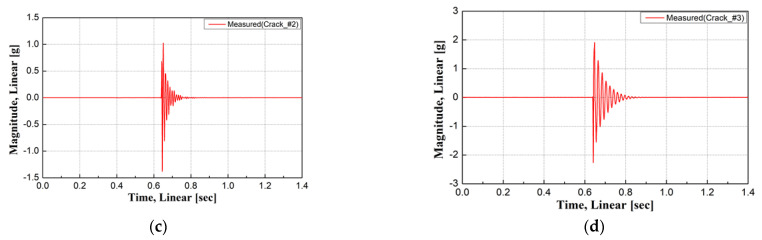
Measurement results of dynamic response function (FRF) for each sleeper. (**a**) Time acceleration (Good); (**b**) time acceleration (Crack #1); (**c**) time acceleration (Crack #2); (**d**) time acceleration (Crack #3).

**Figure 7:**
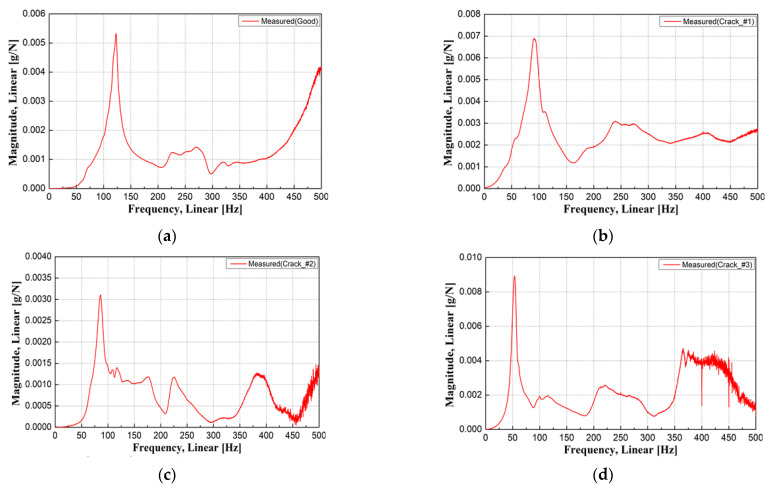
Measurement results of frequency response function (FRF) for each sleeper. (**a**) FRF (good); (**b**) FRF (Crack #1); (**c**) FRF (Crack #2); (**d**) FRF (Crack #3).

Time acceleration was measured using an impact hammer test on damaged concrete sleepers, as shown in Figure 6. The y-coordinate in Figure 7 is the magnitude of acceleration. Figure 7 shows the FRF of each concrete sleeper computed from the impact hammer test. The FRF was used to compute the measured natural frequency for each sleeper.

The FRF was used to measure the natural frequencies of the concrete sleepers, with the natural frequency of the undamaged concrete sleeper found to be approximately 122.96 Hz, as shown in Figure 7a. The natural frequencies of Crack #1 (91.09 Hz), Crack #2 (85.47 Hz), and Crack #3 (53.59 Hz) in the three damaged sleepers, which had similar levels of damage, were measured as shown in Figure 7b–d, experimentally proving that the damage degree of concrete sleepers that are difficult to assess visually can be quantitatively examined using the natural frequency.

Among the damage types, Crack #2 (a crack in the rail seat only) and Crack #1 (a crack in the sleeper center that did not penetrate the ground) exhibited similar natural frequencies. Crack #3 had the lowest natural frequency because it penetrated the bottom of the concrete sleeper core and because there was a partial loss of concrete in the crack in the rail seat, suggesting that the damage degree of concrete sleepers can be estimated according to the natural frequency measured in the field. Furthermore, visual inspection alone was insufficient to assess the structural integrity of the concrete sleepers. In addition, the conditions of nighttime inspections on operating lines made it difficult to secure reliable assessment results for the sleeper conditions through visual inspection.

### 3.2. Dynamic Mass and Stiffness Prediction Using FRF

#### 3.2.1. Dynamic Mass-Prediction Results

According to Esmaeill et al. [5], the impact load intensity has a significant impact on the dynamic resistance. Therefore, the FRF was analyzed based on the impact hammer test measured in the field to understand the effect of state changes of a concrete sleeper on its dynamic mass, and the dynamic mass of the structure was predicted.

**Figure 8:**
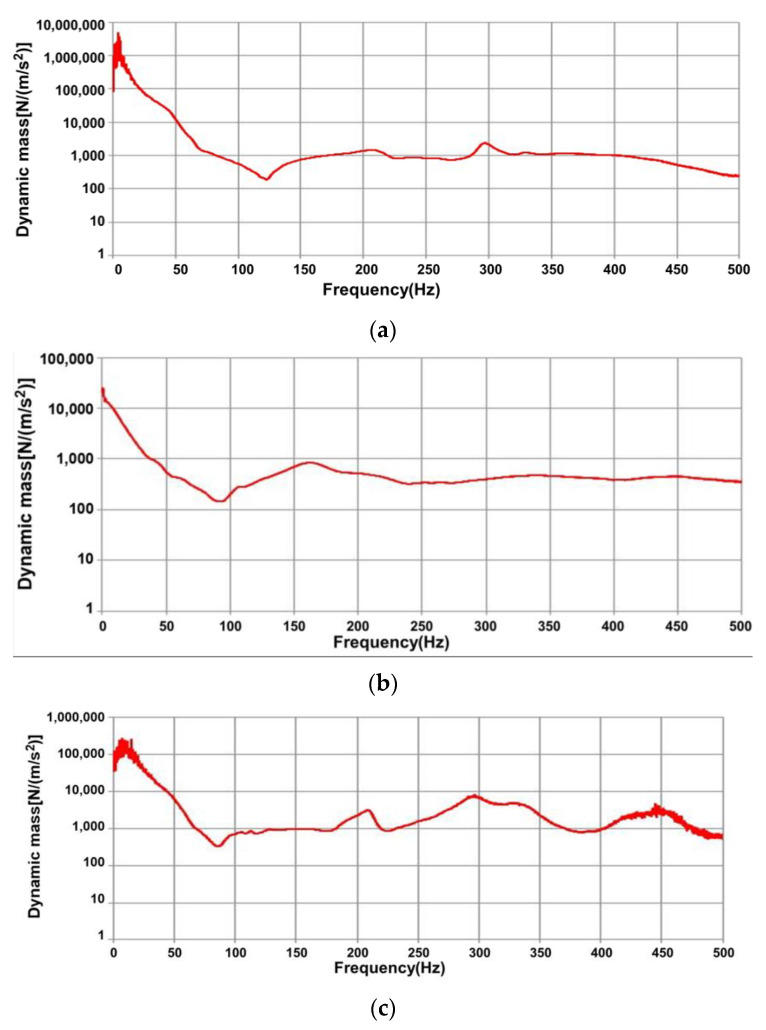

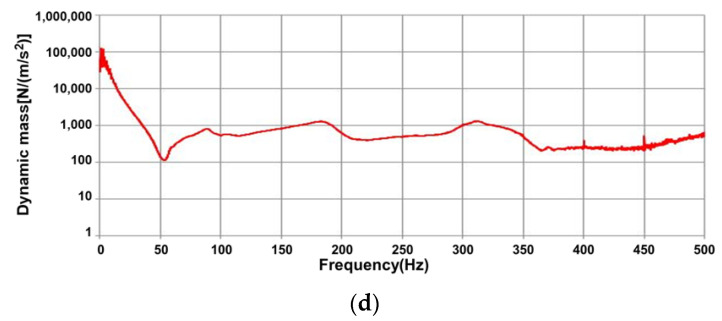
Dynamic mass prediction results. (**a**) Undamaged concrete sleeper; (**b**) Crack #1; (**c**) Crack #2; (**d**) Crack #3.

Figure 8a–d show the dynamic mass of the concrete sleepers as a function of state.

As shown in Figure 8a, when the concrete sleeper is good, the dynamic mass approaches 1. It shows a tendency to converge to 1 × 10^3^ N/(m/s^2^).

In the case of Crack #1, the side section of the sleeper where the accelerometer was installed was completely split in the vertical direction, so the acceleration response was measured separately from the parent body in terms of mass. Figure 8b shows that in the case of Crack #1, where a crack exists in the center of the sleeper, the initial dynamic mass measured by the natural frequency is up to 1 × 10^4^ N/(m/s^2^). It rapidly decreases up to the measured natural frequency of 91.09 Hz and shows a tendency to converge between 1 × 10^2^ N/(m/s^2^) and 1 × 10^3^ N/(m/s^2^).

In the case of Crack #3, the condition was that the crack could be repaired and attached, but it would not contribute to rigidity. Figure 8c shows that when a crack occurs directly below the rail (Crack #2), the initial dynamic mass measured at the natural frequency is 1 × 10^5^ N/(m/s^2^). It decreases up to the measured natural frequency of 85.47 Hz and shows a tendency to converge between 1 × 10^3^ N/(m/s^2^) and 1 × 10^4^ N/(m/s^2^).

Figure 8d shows the case of Crack #3, where a crack penetrates to the bottom in the center of the concrete sleeper and a portion of the concrete cross-section is lost along with the crack directly below the rail. The initial dynamic mass measured at the natural frequency is up to 1 × 10^5^ N/(m/s^2^). It decreases rapidly up to the measured natural frequency of 53.59 Hz and shows a tendency to converge between 1 × 10^2^ N/(m/s^2^) and 1 × 10^3^ N/(m/s^2^).

#### 3.2.2. Dynamic Stiffness Prediction Results for Concrete Sleepers

**Figure 9:**
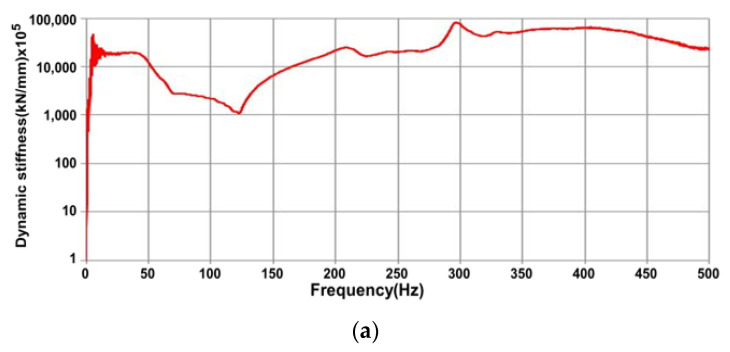

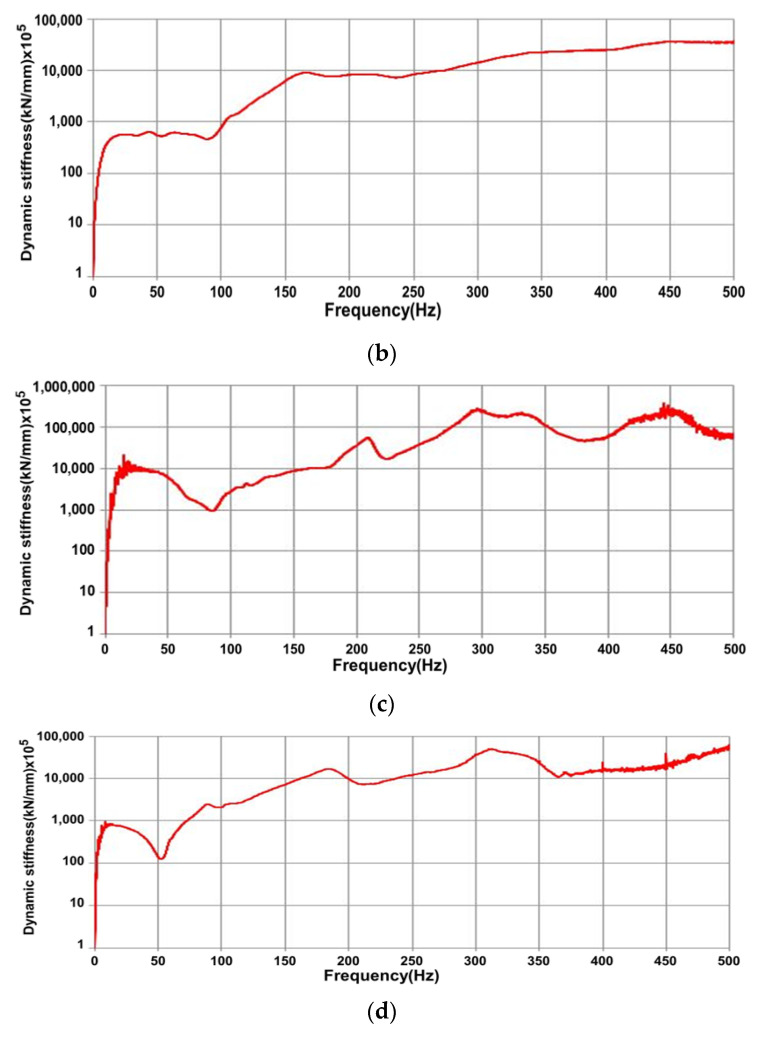
Dynamic stiffness prediction results. (**a**) Undamaged concrete sleeper; (**b**) Crack #1; (**c**) Crack #2; (**d**) Crack #3.

The dynamic stiffness of the concrete sleepers was determined by evaluating the FRF from the impact hammer test measured in the field to understand the effect of state changes of a concrete sleeper on its dynamic stiffness. Figure 9a–d shows the dynamic stiffness of the concrete sleepers.

A sleeper with a crack in the center that did not penetrate the ground (Crack #1), a sleeper with a crack in only the rail sheet (Crack #2), and a sleeper with a crack that penetrated the bottom of the concrete sleeper core and lost part of the concrete from the rail sheet crack (Crack #3) were confirmed to have an effect on the dynamic stiffness. When concrete is damaged, its dynamic stiffness is measured to be low. As shown in Figure 9b,d, the initial dynamic stiffness was measured to be low in Cracks #1 and #3, where critical sleeper cracks occurred. It was possible to predict using FRF that this phenomenon affected the stiffness of concrete sleepers depending on the degree of damage caused to the sleepers. In addition, as shown in Figure 9a,c, it was confirmed that in the case of undamaged concrete sleeper and Crack #2, no direct damage to the concrete sleeper occurred, and the rigidity of the concrete sleeper was not significantly affected.

### 3.3. Numerical Analysis Results

**Figure 10:**
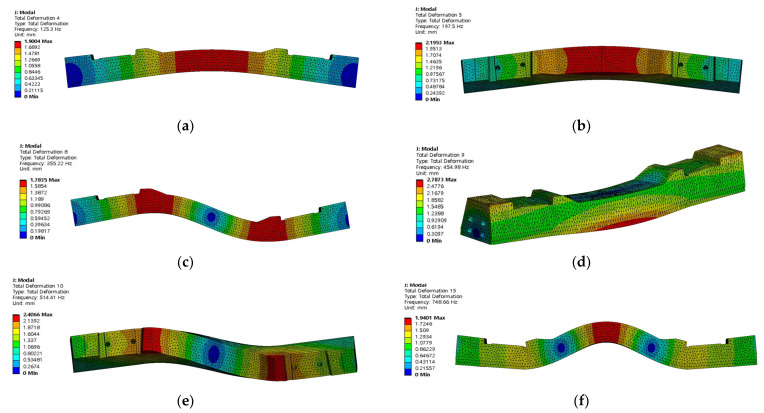
Analysis results (mode shape). (**a**) 1st mode (125.30 Hz); (**b**) 2nd mode (197.50 Hz); (**c**) 3rd mode (355.22 Hz); (**d**) 4th mode (454.98 Hz); (**e**) 5th mode (514.41 Hz); (**f**) 6th mode (748.66 Hz).

A harmonic analysis was performed on the mode with the highest frequency among the six modes obtained through modal analysis. A harmonic analysis was used to perform the numerical analysis, and the mode shape of the concrete sleeper in bending mode was investigated, as shown in Figure 10. The mode analysis showed that the first mode in Figure 10a had a natural frequency of 125 Hz and had a bending deformation characteristic. The second mode in Figure 10b, third mode in Figure 10c, fourth mode in Figure 10d, fifth mode in Figure 10e, and sixth mode in Figure 10f had natural frequencies of 198, 355, 455, 514, and 749 Hz, respectively.

**Figure 11:**
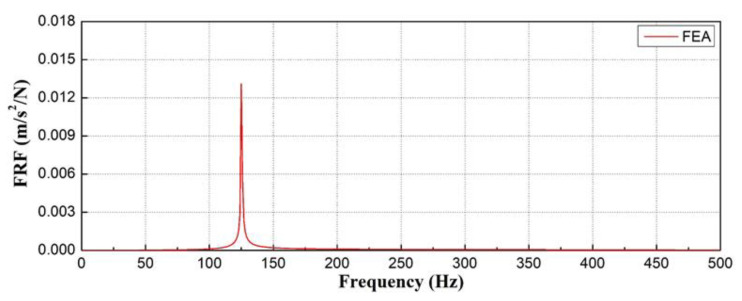
Analyzed natural frequency of concrete sleeper with normal condition.

Figure 11 shows that deformation occurs in the concrete sleepers owing to a bending mode with a natural frequency of 125.30 Hz in the first mode.

### 3.4. Correlation between Concrete Sleeper Damage and Natural Frequency

**Figure 12:**
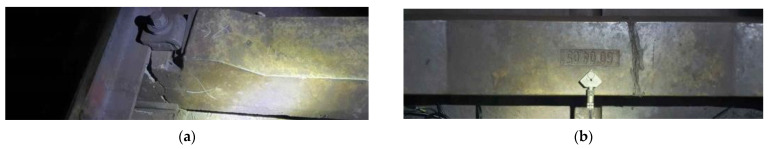
Damage of concrete sleepers. (**a**) Bottom of the rail; (**b**) center of concrete sleeper.

To examine the structural soundness of the numerical analysis and the field-measured condition, the findings of the FRF analysis and the calculation of dynamic mass-and-stiffness values were compared with the field-measured concrete sleepers. Damage occurred mostly in the rail seat and sleeper core of the concrete sleepers measured in this investigation. Figure 12 shows the damaged concrete sleeper. Figure 12a shows damage occurring at the bottom of the rail, and Figure 12b shows damage occurring at the center of the concrete sleeper.

The field impact hammer tests reveal that the change in dynamic mass of concrete sleepers caused by damage was minimal. However, there was a difference in the dynamic stiffness. Differences in the natural frequency developed depending on the damage degree of the concrete sleeper, which is consistent with the findings of Lam and Wong [10]. 

The natural frequency in the bending mode of the undamaged sleeper calculated by numerical analysis was 125.30 Hz, whereas the natural frequency of the undamaged concrete sleeper measured in the field was 122.96 Hz. The error between the two natural frequencies is approximately 1.87%, confirming that the numerical analysis and measurement results are comparable. Since the numerical simulation and field measurement results for the undamaged concrete sleepers were similar, it is inferred that the numerical simulation model accurately captures the behavior of concrete sleepers in the field. Therefore, the applicability of the numerical model and results were validated. Numerical analysis confirmed the natural frequency by modeling a concrete sleeper in good condition. The natural frequencies of intact concrete sleepers and the natural frequencies obtained through the numerical analysis were confirmed to be similar.

**Figure 13:**
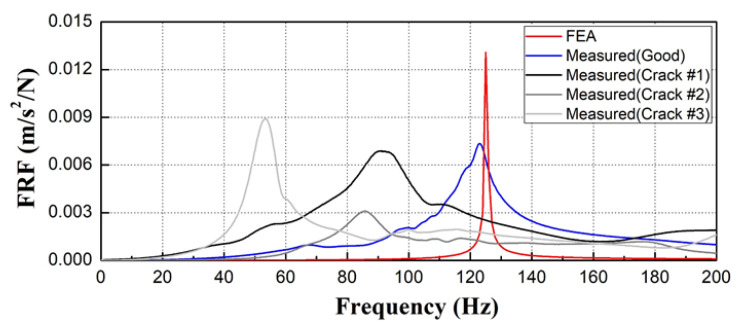
Comparison of modal test and numerical analysis results.

The natural frequencies observed in the damaged concrete sleepers were approximately 27–57% lower than those of the numerical analysis results (125.30 Hz), as illustrated in Figure 13: Crack #1 (91.09 Hz), Crack #2 (85.47 Hz), and Crack #3 (53.59 Hz).

From the impact hammer tests, it can be seen that differences in the natural frequency developed depending on the damage degree of the concrete sleeper. Thus, even though the visible damage of the concrete sleepers is comparable, they may have varying structural soundness values due to unseen damage. Field studies and computational analyses show that a quantitative condition assessment based on measured natural frequencies is attainable using an impact hammer test, which is simple to use in the field.

## 4. Conclusions

This study proposed the impact hammer test as a quantitative assessment method to evaluate the structural soundness of concrete sleepers. It is a portable test method that is easy to apply in practice, and the results are as follows.

The error between the simulated and measured natural frequencies of the undamaged concrete sleeper was approximately 1.87%, confirming the appropriateness of the numerical analysis model. The measured natural frequencies of the damaged concrete sleepers were approximately 27–57% lower than the studied natural frequencies of the undamaged concrete sleeper, demonstrating that the damage to the concrete sleepers had a direct effect on their natural frequency.

The dynamic masses of the natural frequency prediction of the undamaged and damaged concrete sleepers were compared using the impact hammer test in the field, and it was found that the dynamic masses of the damaged concrete sleepers were at least 10 times smaller than that of the undamaged concrete sleeper. The undamaged concrete sleeper exhibited a higher dynamic stiffness than the damaged concrete sleepers, and the natural frequencies estimated for the comparably damaged sleepers (53.59–91.09 Hz) were 1.34–2.29 times lower than the undamaged concrete sleeper (122.96 Hz). These findings show that the dynamic mass and stiffness of damaged concrete sleepers have a lower natural frequency than undamaged concrete sleepers, demonstrating the relationship between concrete sleeper damage and natural frequency values.

The structural integrity of a large number of sleepers can be quickly and easily tested quantitatively by determining their natural frequency in the field using the impact hammer test. In the future, the relationship between the degree of concrete sleeper damage (e.g., crack length, crack width, and crack pattern) and natural frequency values should be further investigated. Classifying concrete sleepers based on their state using natural frequency values recorded in the field will allow these structures to be more effectively maintained. In addition, it is believed that the structural integrity of all concrete sleepers can be quantitatively evaluated through analysis of natural frequencies measured using impact hammer testing.

## Figures and Tables

**Table 1:** Sensitivity of sensors for impact hammer test.

Sensor Type	Impact Hammer	Accelerometer		
Channels	Ch.1	Ch.2	Ch.3	Ch.4
Sensitivity	0.2301 mV/N	1024.0 mV/g	1018.0 mV/g	993.2 mV/g

**Table 2:** Load-response relationship in the vibration domain.

Response	Inverse FRF	Standard FRF
Acceleration	$F\left(\omega \right)/\ddot{u}\left(\omega \right)$	$\ddot{u}\left(\omega \right)$/$F\left(\omega \right)$
Velocity	$F\left(\omega \right)/\dot{u}\left(\omega \right)$	$\dot{u}\left(\omega \right)$/$F\left(\omega \right)$
Displacement	$F\left(\omega \right)$/*u*(ω)	*u*(ω)/$F\left(\omega \right)$

**Table 3:** Relationship between FRF and dynamic mass and dynamic stiffness.

$\log\left|FRF\right|$	Dynamic Mass (m)	Dynamic Stiffness (k)
$\log\left|\ddot{u}\left(\omega \right)/F\left(\omega \right)\right|$	$\frac{1}{m}-\log\left(m\right)$	$\frac{\omega^{2}}{k}-2\log\left(\omega \right)-\log\left(k\right)$
$\log\left|\dot{u}\left(\omega \right)/F\left(\omega \right)\right|$	$-i\frac{1}{\omega m}-\log\left(m\right)-\log\left(\omega \right)$	$i\frac{\omega }{k}-\log\left(\omega \right)-\log\left(k\right)$
$\log\left|u\left(\omega \right)/F\left(\omega \right)\right|$	$-\frac{1}{\omega^{2}m}-\log\left(m\right)-2\log\left(\omega \right)$	$\frac{1}{k}-\log\left(k\right)$

**Table 4:** Properties of FE model.

Items	Properties		
Rail weight (kg/m)	60		
Sleeper type	Post-tensioning Sleeper		
Sleeper size (mm)	212 × 240 × 2300		
Compressive strength (MPa)	59		
Post-tensioning steel bar	Tensile strength (MPa)	Diameter (mm)	Length (mm)
	1230	Ø9.2	2240
Post-tensioning force (kN/EA)	66.1		
Spring stiffness of rail pad (kN/mm)	50		
Spring stiffness of under sleeper pad (kN/mm)	10		
Spring stiffness of rubber boot (kN/mm)	2000		

## Data Availability

Data are contained within the article.
